# A Comprehensive Molecular and Epidemiological Characterization of Influenza Viruses Circulating 2016–2020 in North Macedonia

**DOI:** 10.3389/fmicb.2021.713408

**Published:** 2021-10-21

**Authors:** Maja Kuzmanovska, Golubinka Boshevska, Elizabeta Janchevska, Teodora Buzharova, Milica Simova, Aneta Peshnacka, Gordana Nikolovska, Dragan Kochinski, Radica Stoleska Ilioska, Kristina Stavridis, Vladimir Mikikj, Gordana Kuzmanovska, Shaban Memeti, Icko Gjorgoski

**Affiliations:** ^1^Laboratory of Virology, Institute of Public Health, Skopje, North Macedonia; ^2^Faculty of Natural Sciences and Mathematics, Skopje, North Macedonia

**Keywords:** influenza, ILI sentinel surveillance system, SARI sentinel surveillance system, A(H1N1)pdm09, A(H3N2), molecular evolution, phylogeny

## Abstract

Influenza viruses know no boundaries, representing an example of rapid virus evolution combined with pressure exerted by the host’s immune system. Seasonal influenza causes 4–50 million symptomatic cases in the EU/EEA each year, with a global death toll reaching 650,000 deaths. That being the case, in 2014 North Macedonia introduced the sentinel surveillance in addition to the existing influenza surveillance in order to obtain more precise data on the burden of disease, circulating viruses and to implement timely preventive measures. The aims of this study were to give a comprehensive virological and epidemiological overview of four influenza seasons (2016–2020), assess the frequency and distribution of influenza circulating in North Macedonia and to carry out molecular and phylogenetic analyses of the hemagglutinin (HA) and neuraminidase (NA) genes of influenza A(H1N1)pdm09, A(H3N2) from ILI and SARI patients. Our results showed that out of 1,632 tested samples, 46.4% were influenza positive, with influenza A(H1N1)pdm09 accounting for the majority of cases (44%), followed by influenza B (32%) and A(H3N2) (17%). By comparing the sentinel surveillance system to the routine surveillance system, we showed that the newly applied system works efficiently and gives great results in the selection of cases. Statistically significant differences (*p* = < 0.0000001) were observed when comparing the number of reported ILI cases among patients aged 0–4, 5–14, 15–29, and 30–64 years to the reference age group. The phylogenetic analysis of the HA sequences unveiled the resemblance of mutations circulating seasonally worldwide, with a vast majority of circulating viruses belonging to subclade 6B.1A. The PROVEAN analysis showed that the D187A substitution in the receptor binding site (RBS) of the A(H1N1)pdm09 HA has a deleterious effect on the its function. The A(H3N2) viruses fell into the 3C.2a and 3C.3a throughout the analyzed seasons. Molecular characterization revealed that various substitutions in the A(H3N2) viruses gradually replaced the parental variant in subsequent seasons before becoming the dominant variant. With the introduction of sentinel surveillance, accompanied by the advances made in whole-genome sequencing and vaccine therapeutics, public health officials can now modify their approach in disease management and intervene effectively and in a timely manner to prevent major morbidity and mortality from influenza.

## Introduction

Influenza viruses know no boundaries, representing an example of rapid virus evolution combined with pressure exerted by the host’s immune system, enabling them to circulate between species and occasionally cross the interspecies barriers. Owing to the negative-stranded segmented RNA genome, influenza viruses constantly undergo spontaneous mutations resulting in small changes in their antigenic properties year to year (Guldemir et al., 2019). These high mutation rates allow for the evasion of immunity. An emergence of an influenza variant with new antigenic properties, due to the possibility of reassortment of their segmented genomes, may increase the infectivity or pathogenicity of the virus and lead to expanded tissue tropism, producing pandemic strains that are antigenically novel but otherwise well adapted to humans (Krammer and Palese, 2013; Lee et al., 2015; Baranovich et al., 2017; Hutchinson, 2018). Two surface glycoproteins of the influenza virus, hemagglutinin (HA) and neuraminidase (NA), are of major importance in both clinical and biological terms. Changes in these two proteins may sometimes lead to the acquisition of glycosylation sites, which is believed to efficiently generate antigenic variants and play a critical role in virus evolution (Gallagher et al., 1988; Igarashi et al., 2010). The antigenic sites around the receptor binding site (RBS) of the HA protein, harbor most of the newly emerged mutations leading to antigenic drift. As one of the major threats on the global health care system, due to its continued potential to cause pandemics, the wide-ranging impact of influenza presents an increasing challenge for prevention and control (Fischer et al., 2015). While influenza A viruses infect both humans and animals, influenza B viruses circulate primarily in humans. The clinical symptoms associated with influenza B virus infections are generally milder compared to those of the influenza A virus. Therefore, pandemic influenza is usually caused by the influenza A virus, due to its rapid antigenic variation, strong replication capacity, and transmission ability associated with genetic reassortment (Chan et al., 2010; White and Lowen, 2018). Seasonal influenza causes 4–50 million symptomatic cases in EU/EEA each year, and 15,000–70,000 European citizens die every year of causes associated with influenza (European Centre for Disease Prevention and Control, 2021), while the death toll in global proportions reaches up to 650,000 deaths (World Health Organization, 2017). Despite the often-short duration of the illness, the yearly economic and healthcare burden of influenza is substantial. Thereby, the all-year-round monitoring of novel influenza mutations and reassortments is an essential activity carried out within the framework of the Global Influenza Surveillance and Response System (GISRS), allowing time to track the appearance of variants of the virus with its modified virological, molecular and/or antigenic properties. Following the example in many countries which have developed systemic influenza surveillance programs after the 2009 pandemic (Soebiyanto et al., 2015), North Macedonia introduced the sentinel surveillance in addition to the existing routine influenza surveillance in order to obtain more precise data on the burden of disease, intensity, circulating viruses and ultimately to implement timely preventive measures. The aims of this study were to give a comprehensive virological and epidemiological overview of the past four influenza seasons (2016–2020), to assess the frequency and distribution of the influenza virus types and subtypes circulating in North Macedonia as well as to carry out molecular and phylogenetic analyses of the HA and NA gene sequences of influenza A(H1N1)pdm09, A(H3N2), B/Victoria and B/Yamagata from patients from the sentinel surveillance.

## Materials and Methods

In the 2014/2015 season, with support from the South East European Center of Infectious Diseases Surveillance and Control–SECID and in relation to the project: “Surveillance and response to avian and pandemic influenza,” Grant No. 5U51IP000841-03 funded by the Center of Disease Control and Prevention (CDC), the Institute of Public Health (IPH) initiated a project for sentinel surveillance of Influenza like Illnesses (ILI) and Acute Respiratory Infection (ARI), as well as Severe Acute Respiratory Infections (SARI). A total number of 1,632 respiratory samples (nasal and throat swabs) were prospectively collected throughout the past four influenza seasons (2016–2020) through our ILI/ARI and SARI sentinel surveillance system. The ILI/ARI sentinel surveillance involves a limited number of selected primary healthcare sites (outpatients), while the SARI sentinel surveillance is focused on hospitals and involves only hospitalized patients. In North Macedonia the ILI and SARI cases are followed both epidemiologically and virologically, whereas the ARI cases are monitored only epidemiologically. A total of 19 ILI/ARI sites were included, covering primary care settings in 8 out of 10 regions, with population catchment of over 2% in each region. Six SARI sentinel sites were involved, of which five were in Skopje–three University clinics and two hospitals and one hospital in Prilep. The sentinel sites from the ILI/ARI network work throughout the whole year, whereas the samples collection from the SARI sentinel sites is carried out from week 40 to week 20 of the following year. The sampling was carried out in the first 2–7 days of the symptoms onset. All clinical samples were transported in virus transport medium (VTM) (COPAN Diagnostics, Inc., Murrieta, CA, United States), accompanied by patients’ anonymous forms, including age, gender, sampling date, place of residence and symptoms. As an inclusion criteria, the patients should meet the clinical case definition for ILI (ARI with fever ≥38°C and cough with onset within the last 10 days) or SARI (SARI with fever ≥38°C, cough with onset within the last 10 days and required hospitalization). The patients that did not meet the clinical case definition for ILI and SARI were excluded from the study. For further analysis of the molecular evolution of the influenza A subtypes H1N1pdm09 and H3N2, as well as influenza B subtypes Yamagata and Victoria we collected all HA and NA sequences of both subtypes uploaded in GISAID (Global Initiative on Sharing Avian Influenza Data)^1^ by our laboratory and the WHO Collaborative Centers in London and Atlanta in the period 2016–2020. Influenza vaccine strains and representative strains published on the WHO collaborating center website (World Health Organization, 2018) were used as references. We acknowledge the authors and submitting laboratories to all datasets used in this study.

### Ethics Statement

Informed consent for influenza detection, typing and molecular characterization was not required since all ILI and SARI patients were analyzed anonymously in accordance with the Protocol for investigation of acute respiratory illness outbreaks of unknown etiology by WHO (World Health Organization, 2011) translated and adapted for North Macedonia as Protocol for influenza outbreak investigation and other acute respiratory diseases.

### Detection and Molecular Characterization of Influenza Viruses

Viral RNA extractions from clinical samples from 2016 to 2018 were performed with the use of the RNeasy Mini Kit according to the manufacturer’s instructions (Qiagen, Hilden, Germany). From the beginning of 2018/2019 season we started using the instrument MagCore16 for automatic extraction according to the manufacturer’s instructions (RBC Bioscience, New Taipei City, Taipei). A panel of RT-qPCRs were performed to detect respiratory viruses, including influenza A (with subtyping H1N1pdm09 and H3N2) and B (World Health Organization, 2011). The primers and probes used for the assays were donated by the CDC. The CDC influenza virus RT-PCR A/B typing panel (cat. No FluRUO-01) was used for the detection of influenza A and B, the CDC influenza virus RT-PCR H1pdm09/H3 subtyping panel (cat. No FluRUO-09) was used for the typing of influenza A/H1pdm09 and A/H3, whereas the CDC RT-PCR influenza B genotyping panel (cat. No FluRUO-11) was utilized for the detection of B/Yamagata and B/Victoria lineages. Reactions were performed with 25 μl Ambion Ag-Path Master Mix (Life Technologies, United States) using TaqMan Chemistry on Quant Studio 5 (Applied Biosystems, Foster City, CA, United States) and CFX96 (Bio-Rad, Hercules, CA, United States).

### Sanger Sequencing of Influenza Hemagglutinin and Neuraminidase Genes

cDNA was made from each RNA sample using ThermoScript RT-PCR System for First-Strand cDNA Synthesis (Invitrogen, Carlsbad, CA, United States), with Uni12 primers (Hoffmann et al., 2001). Two microliter of the cDNA was amplified in a 50 μl reaction volume using the Platinum Taq DNA High Fidelity Polymerase kit (Invitrogen, Carlsbad, CA, United States) and specific gene primers for HA and NA of A(H1N1)pdm09 and A (H3N2) (Deng et al., 2015). Purified PCR products were sequenced using the BigDye Terminator Cycle-Sequencing kit (Applied Biosystems, Foster City, CA, United States) on ABI Prism 310 Genetic Analyzer (Applied Biosystems, Foster City, CA, United States).

### Nanopore Sequencing

RNA was amplified simultaneously using a universal primer set adapted to the conserved 3′ and 5′ segment ends for full-length amplification of all influenza A viruses (Hoffmann et al., 2001) using Superscript III One-Step RT-PCR with Platinum Taq (Invitrogen, Carlsbad, CA, United States). The PCR conditions were as described in an article by King (King et al., 2020). After amplification, the samples were purified with AMPure XP Magnetic Beads (Beckman Coulter, Fullerton, CA, United States) in a × 0.65 sample volume to bead volume ratio. Quantification was conducted with the Qubit 4 Fluorometer (Thermo Fisher Scientific).

Following the manufacturer’s instructions, the Native Barcoding kit (cat. no. ONT EXP-NBD104) and the Ligation kit (cat. no. ONT SQK-LSK109) were used. After library preparation, the pooled samples were loaded onto a FLO-MIN106 R9.5 flow cell. An 8-h run was conducted with standard settings.

### Analysis of MinION Sequencing Data

Real time basecalling was performed with MinKNOW and its integrated Guppy v3.0.4 software (ONT) to produce fast5 and fastQ files. The minimum q-score a read must attain to pass qscore filtering is 7, roughly corresponding to a basecall accuracy of 85%. After basecalling, we used EPI2ME and its tool WIMP for rapid species identification and full QC metrics to have an insight in the runs’ performance including number of reads, read length distribution and quality scores. The quality checked reads were demultiplexed and trimmed for adapters using ONT Guppy Barcoding Software v3.1.5 + 781ed575. The mapping, alignment, consensus production and variant calling was carried out with the CLC Genomic Workbench version 20.0.4 (Qiagen, Hilden, Germany). Additionally, we used several different GALAXY tools for data analysis.^2^ Firstly, we concatenated datasets and did a fast QC check, followed by mapping with minimap2. For variant calling we used OCOCO, an online consensus caller, while MAAFT was utilized for a high-speed multiple sequence alignment.

### Phylogenetic Analyses

Nucleotide alignments were constructed using ClustalW (Larkin et al., 2007) followed by alignment to codon position in Molecular Evolutionary Genetics Analysis (MEGA; Kumar et al., 2008). A maximum likelihood phylogenetic tree with General time reversible model (GTR) was inferred using MEGA and the reliability of the tree topology was assessed by bootstrap analysis with 1,000 replications. For better graphic representation of the phylogenetic trees, Fig Tree was used. For the phylogenetic analysis, the HA amino acid sequences of the reference viruses with known genetic group identities and representing different countries of Europe season were retrieved from the GISAID database (see text footnote 1). Additionally, we included the sequences of the vaccine strains from the respective years. Such robust tree will allow us to determine the phylogenetic relationship of HA genes of influenza A(H1N1)pdm09 and A(H3N2) circulating in different seasons in North Macedonia.

### Sequence Analysis

For the molecular evolution analysis, the sequences were compiled and edited using Bioedit and MEGA. Multiple sequence alignments for A(H1N1)pdm09 and A(H3N2) HA and NA sequences were carried out using ClustalW Algorithm. For the sequence analysis the recommended numbering scheme was used for the HA subtypes (Burke and Smith, 2014). The identified amino acid substitutions within the A(H1N1)pdm09 genes of the Macedonian viruses were mapped to previously reported HA antigenic sites (Sa, Sb, Ca1/2, and Cb) (Caton et al., 1982) and NA antigenic sites (Maurer-Stroh et al., 2009).

### Prediction of Mutations Effect on Hemagglutinin Function

The PROVEAN (Protein Variation Effect Analyzer) software tool enabled us to predict the possible effect of identified amino acid substitution on the biological function of the HA protein.^3^ To provide binary predictions, the cutoff for PROVEAN scores was set to −2.5 for high balanced accuracy (Choi and Chan, 2015).

### Statistical Analysis

Microsoft Excel 2016 for Windows 10 and OpenEpi were used for statistical data analyses. The χ^2^ test and Fisher’s exact test were used to compare frequencies between different groups. A *p* value of >0.05 was considered significant. Relative risk (RR) was used to estimate the strength of association between two groups or to show the difference in incidence between the control and reference group. RR >1 means that the risk of the outcome is increased by the exposure, which is a “risk factor.” The results are presented in tabular and graphical form.

## Results

### Temporal Distribution of Influenza

**FIGURE 1:**
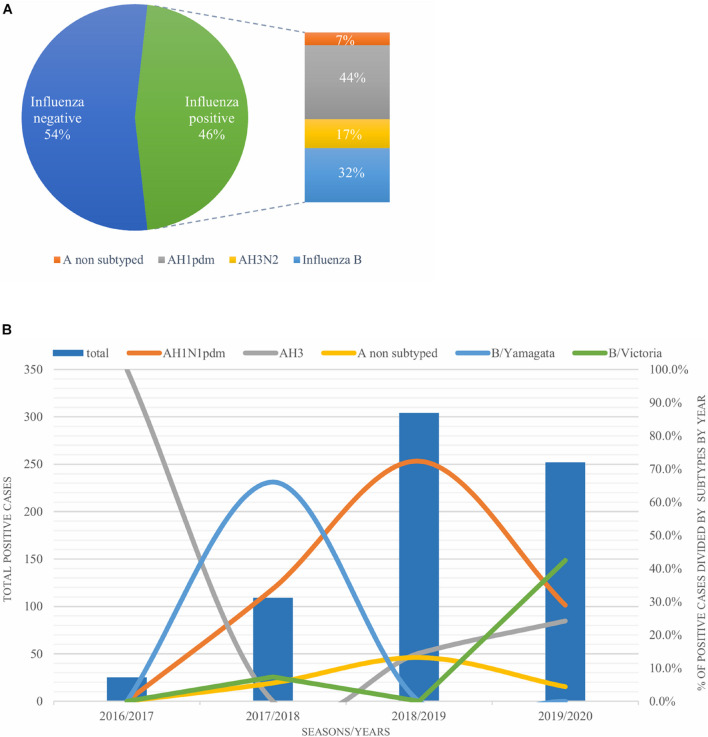
**(A)** Percentage of influenza types and subtypes from ILI and SARI surveillance (2016–2020). **(B)** The total number of influenza positive samples per year is presented by blue bars. Annual percentages of reported positive cases for influenza A subtypes H1N1pdm09 (orange), H3N2 (gray) and influenza B/Victoria (green) and B/Yamagata (blue) are also presented.

**FIGURE 2:**
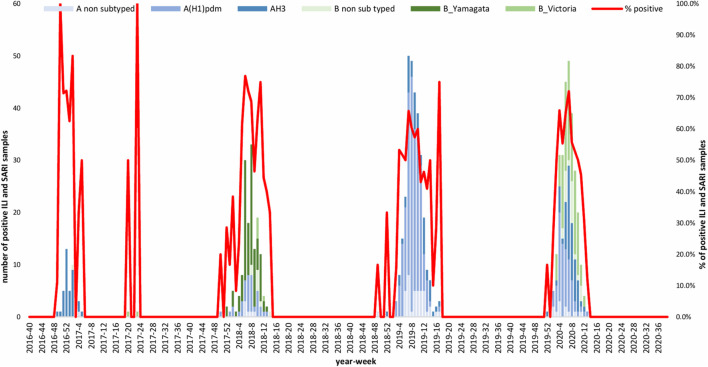
Graphical representation of ILI and SARI positive cases divided by week and subtype.

A total of 1,632 samples from the ILI and SARI sentinel surveillance were tested, of which 46.4% (*n* = 758) were influenza positive cases. Influenza A(H1N1)pdm09 was predominant, reaching 44% of the total number of positive samples, followed by influenza B (32%), A(H3N2) (17%) and A non-subtyped (7%) (Figure 1A). Throughout the analyzed period, we had quite dynamic influenza seasons. During season 2016/2017, influenza A(H3N2) was predominant in North Macedonia, whereas in 2017/2018 influenza B/Yamagata reached the highest frequency with up to 72% of the total positive cases. During this season within the group of influenza B viruses, B/Yamagata and B/Victoria co-circulated in North Macedonia with frequency of 90 and 10%, respectively. In the 2018/2019 season, the percentage of influenza A(H1N1)pdm09 increased rapidly reaching a frequency of 72%, while in 2019/2020 influenza A and B co-circulated, 53 and 47%, respectively. Among the influenza A positive samples, A(H1N1)pdm09 was once again the predominant type (26.5%), followed by A(H3N2) (22.2%) and A non-subtyped. Contrary to season 2017/2018, in 2019/2020 the predominating subtype was B/Victoria (100%) (Figure 1B). A clear graphical representation depicts the epidemiological curve of positive samples, divided by week, from the ILI and SARI surveillance (Figure 2). For the past four seasons, the highest influenza activity was reported from the end of December (week 52) to the middle of April (week 15), with a peak in mid-February (week 8), followed by a sharp decline in the number of positive cases. In general, the percentage of positive cases is corresponding to the number of tested and positive cases throughout the seasons, with the exception of the 2016/2017 season which was characterized with a low number of tested as well as positive samples. The sentinel surveillance system in North Macedonia works as efficiently as the routine surveillance system, with both epidemiological curves coinciding throughout the analyzed seasons (Supplementary Figure 1). The highest number of reported ILI cases was among patients aged 30–64 years (Supplementary Figure 2) and this difference was statistically significant when compared to the reference age group (65+) (*p* = < 0.0000001). Furthermore, among the reported ILI cases, we observed statistically significant differences between the reference age group and the age groups 0–4, 5–14, and 15–29 (*p* = < 0.0000001) (Table 1).

**TABLE 1:** Statistically significant differences between all analyzed age groups compared to the respective population under surveillance.

Age group	ILI cases	Non-ILI cases	ILI avg population	RR	CI	*P*-value
0–4	543	3,488	4,031	2,235	1.964–2.543	<0.0000001
5–14	742	6,286	7,028	1,752	1.548–1.982	<0.0000001
15–29	678	6,459	7,137	1,576	1.391–1.787	<0.0000001
30–64	1,139	14,809	15,948	1,185	1.054–1.332	<0.0000001
65+	341	5,317	5,658	Ref		

Additionally, we compared the number of positive ILI and SARI samples with the predominant virus type of each season, in order to investigate the possible connection between a particular virus subtype and the severity of the disease (Table 2). However, we could not make such distinction. The percentage of positive samples from the ILI and SARI surveillance was similar for all subtypes throughout the analyzed seasons.

**TABLE 2:** Comparison of the number of positive ILI and SARI samples with the predominant virus type of each consecutive influenza season.

Type/subtype	2016/2017		2017/2018		2018/2019		2019/2020	
	ILI	SARI	ILI	SARI	ILI	SARI	ILI	SARI
A unsubtyped	0	0	0	5.10%	12.90%	9.70%	4.40%	4.60%
A/H1N1pdm09	0	0	19%	25.60%	74.70%	76.60%	19.20%	35.30%
A/H3N2	100%	100%	0	0	14%	13.70%	29.30%	20.90%
B/Yamagata	0	0	73.80%	61.50%	0	0	0	0
B/Victoria	0	0	7.90%	7.70%	0	0	47.50%	39.20%

We differentiated between the number of reported, tested and positive SARI cases. The percentage of tested in relation to reported cases has notably increased, from 25% for 2017/2018 to 45% for the 2019/2020 season. The positivity rate among the tested samples varied, from 25% for 2016/2017 season, 56% for 2017/2018, 40 and 44%, respectively, for the last two seasons (Supplementary Figure 3).

A total of 371 sequences were analyzed: 68 H1 sequences, 79 H3 sequences, 66 N1 sequences. Seventy-eight N2 sequences, 18 B/Yamagata HA and NA sequences and 22 B/Victoria HA and NA sequences. The highest number of sequences was obtained during the 2018/2019 influenza season, due to the highest number of positive samples. Table 3 gives an overview of the number of influenza A and B positive samples and number of HA/NA sequences analyzed in this study.

**TABLE 3:** Total number of influenza A and B positive samples and number of HA/NA sequences analyzed in this study.

Year	H1N1pdm09				H3N2			
	Number of positive samples	Number of sequences analyzed		Vaccine strain	Number of positive samples	Number of sequences analyzed		Vaccine strain
		H1	N1			H3	N2	
2016/2017	1	1	1	A/California/07/2009	25	23	23	A/Hong Kong/4801/2014
2017/2018	37	11	11	A/Michigan/45/2015	0	0	0	A/Hong Kong/4801/2014
2018/2019	220	42	40	A/Michigan/45/2015	44	26	25	A/Singapore/INFIMH-16-0019/2016
2019/2020	73	14	14	A/Brisbane/02/2018	71	30	30	A/Kansas/14/2017

**Year**	**B/Yamagata**				**B/Victoria**			
	**Number of positive samples**	**Number of sequences analyzed**		**Vaccine strain**	**Number of positive samples**	**Number of sequences analyzed**		**Vaccine strain**

2016/2017	0	0	0	/	0	0	0	B/Brisbane/60/2008
2017/2018	105	18	18	/	8	4	4	B/Brisbane/60/2008
2018/2019	0	0	0	B/Phuket/3073/2013	0	0	0	B/Colorado/06/2017
2019/2020	0	0	0	B/Phuket/3073/2013	130	18	18	B/Colorado/06/2017

*For the evolutionary analysis, complete HA and NA sequences for H1N1pdm09, H3N2, B/Victoria and B/Yamagata viruses were downloaded from GISAID.*

### Phylogenetic Tree Analysis

**FIGURE 3:**
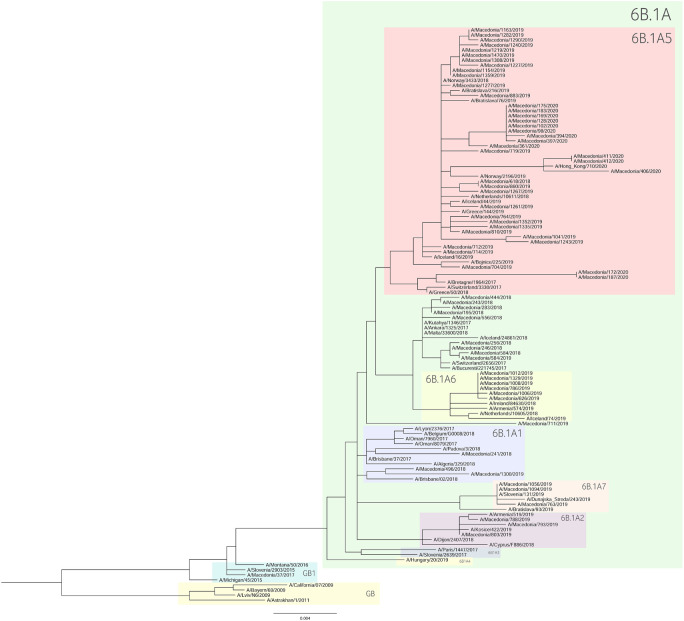
A(H1N1)pdm09 HA phylogenetic tree.

**FIGURE 4:**
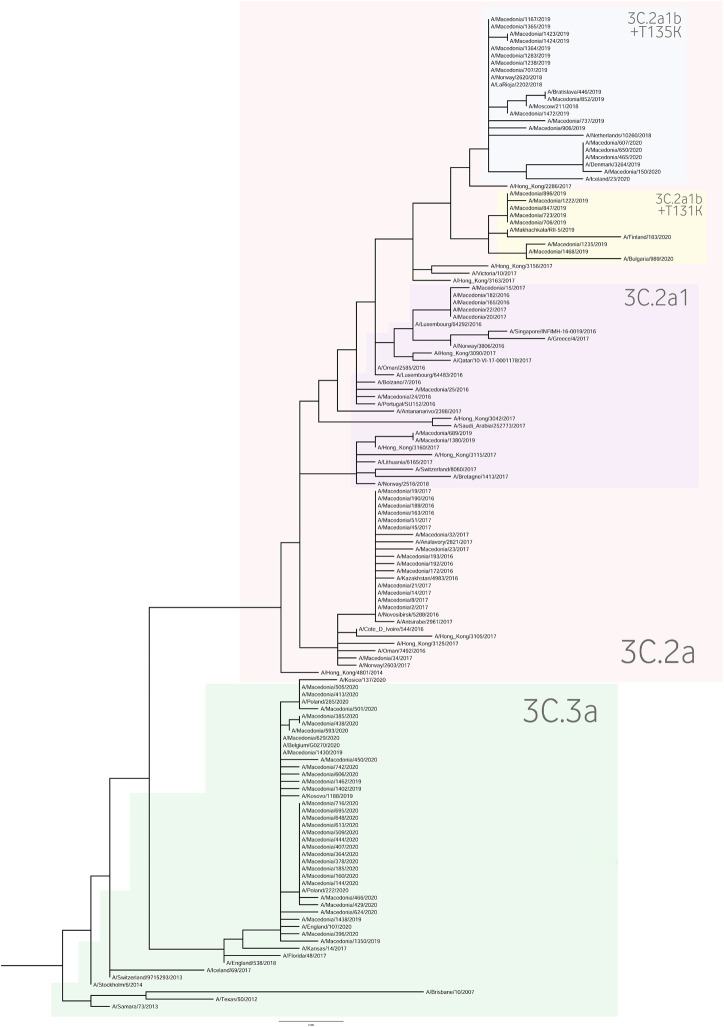
A(H3N2) HA phylogenetic tree.

For the construction of the phylogenetic trees, we used the HA sequences of A(H1N1)pdm09 and A(H3N2) viruses along with the vaccine strain sequences and additional reference sequences (Figures 3, 4, respectively). Twenty percent of the total number of positive A(H1N1)pdm09 samples and 54.1% of the A(H3N2) viruses were sequenced and included in the phylogenetic analysis. During the 2016/2017 season all of the sequenced H3N2 HA genes fell into the 3C.2a clade or the 3C.2a1 subclade. The 3C.2a clade is defined by the amino acid substitutions N121K, S144K, F159Y, K160T (resulting in the gain of a potential glycosylation site), N225D and Q311H in HA1. The subclade 3C.2a1, characterized by the amino acid substitution N171K in HA1 and I77V and G155E in HA2 was defined in 2016 as a genetic group that was increasing in prevalence at that time and has continued to increase in prevalence. Of the total number of sequenced HA genes 78.3% belonged in the 3C.2a clade, while 21.7% the sequences fell into 3C.2a1 subclade and harbored additional substitutions N121K, I140M, and G142R in HA1 and G150E in HA2. The phylogenetic analysis showed that all influenza A(H1N1)pdm09 HA sequences from 2017/2018 belonged to the 6B.1 clade encoding the substitutions S74R, S164T, and I295V in HA1. This clade is represented by the A/Michigan/45/2015 reference strain, which was included in the 2017–2018 vaccine. It is notable that 81.81% had the substitution T120A in HA1. During the 2018–2019 influenza season, the vast majority of A(H1N1)pdm09 viruses belonged to the HA phylogenetic subclade 6B.1A which evolved from clade 6B.1. Within subclade 6B.1A genetic clusters of viruses encoding a range of HA1 amino acid substitutions have emerged with the major groups being: 6B.1A2, with HAs defined by the substitutions L233I in HA1 and V192A in HA2 (V520A), 6B.1A5, with HAs defined by N129D, T185I, and N260D in HA1, 6B.1A6, with HAs defined by T120A in HA1 and 6B.1A7, with HAs defined by K302T in HA1 and I76M, N169S, and E179D in HA2. From our A(H1N1)pdm09 sequenced HA genes 59.5% belonged to the 6B.1A5 subclade, whereas 21.4% fell into the 6B.1A6 group. Small number of HA sequences clustered in the 6B.1A2 and 6B.1A7 subclades. The HA genes of H3N2 viruses have fallen within clades 3C.3a and 3C.2a but the genetic diversity of the HA genes during 2018/2019 flu season has increased and new subclades and genetic groups have been defined. All share a N225D substitution in HA1. Namely, subclade 3C.2a1b, with the substitutions E62G, K92R, N121K, a reversion R142G, N171K, and H311Q in HA1 and I77V, G155E in HA2, has predominated. Within this subclade there are two main genetic groups defined by T128A and T135K in HA1 both resulting in the loss of glycosylation motifs, T131K in HA1 and V200I in HA2. Clade 3C.3a viruses are defined by the substitutions T128A, A138S, and R142G in HA1. The HA genes of recent clade 3C.3a viruses encode the substitutions L3I, S91N, N144K (resulting in the loss of a potential glycosylation site), F193S and K326R in HA1, and D160N in HA2. Twenty three percent of the HA genes belonged in clade 3C.3a. The others were in subclade 3C.2a1b, 26.9% falling into a genetic subgroup with HA genes encoding a T131K substitution and 46.15% into a genetic group encoding the substitutions T128A and T135K in HA1, both of these substitutions causing a loss of glycosylation sites in HA1. All of the sequenced A(H1N1)pdm09 HA genes from the 2019/2020 season were within subclade 6B.1A, more specifically 6B.1A5. The genetic clusters which have emerged from the 6B.1A subclade and the amino acid substitutions were explained in detail in the previous paragraph. The phylogenetic analysis of H3N2 viruses showed that 86.67% of the sequenced viruses belonged to 3C.3a. The rest of the viruses (13.3%) were in subclade 3C.2a1b, or subclade 3C.2a1b + T135K encoding the HA1 substitutions A138S, G186D, D190N, F193S, and S198P viruses with these substitutions have now become a major group.

### Genetic Characterization of A(H1N1)pdm09 Viruses

#### Molecular Evolution of H1 Protein

The amino acid sequences of HA of A(H1N1)pdm09 were compared to the vaccine strain A/California/07/2009 (Table 4). The alignment of the 68 HA sequences showed high homology between the viruses circulating during influenza seasons 2016–2020 and A/California/07/2009. From season 2016/2017 we had only one HA sequence, therefore these results are not representative for the season. In total, 32 amino acid substitutions were detected in the HA protein of which 25 were in HA1 or its globular head and 7 in H2 (stem region). Ten of the identified substitutions were found in the major antigenic sites such as E154K, S162N, K163Q, and S164T in Sa, S203T in Ca1 and S185T/I, D187A, and Q189E in Sb. Almost all of the amino acid residues within the RBS were highly conserved, detecting only one substitution D187A in the 190-loop. The substitutions E154K, S162N, K163Q, and S164T showed complete dominance, reaching a frequency of 100% in the analyzed seasons. On the other hand, the frequency of S183P was significantly higher (*p* = 0.00001) in season 2018/2019 (97.62%) vis-a-vis season 2017/2018 (18.18%). The frequency of S185T progressively decreased throughout the seasons 2016–2020 (100, 90.91, 38.10, and 14.29%, respectively), whereas the percentage of S185I significantly increased (*p* = 0.0002) from 9.09% in the 2017/2018 season to 85.71% in 2019/2020. The D187A and Q189E substitutions were present only in the 2019/2020 season with a frequency of 64.29%. Viruses circulating during the four analyzed seasons were generally stable, with several new variants being detected. Most of the viruses isolated in the period 2016–2020 (95–100%) were carrying fixed mutations that have been established in previous seasons such as S74R, P83S, S84N, D97N, E154K, S162N, K163Q, S185T, I216T, A256T, K283E, I295V, I321V in the HA1 subunit and E374K, S451N, and E499K in the HA2 subunit. In season 2017/2018 the two most notable substitutions were T120A (81.82%) and S185T (90.91) in Sb. During 2018/2019 season we detected several different substitutions with high frequencies, such as N129D (57.14%), S185I (61.9%), S185T (38.10%), and N260D (59.52%), while only minority of the viruses were carrying T120A (16.67%), L233I (7.14%), I404M (9.52%), K504R (9.52%), E506D (7.14%), and V520A (9.52%). In season 2019/2020 we noticed the highest number of newly emerged substitutions, such as K130N (35.71%), D187A (64.29%), Q189E (64.29%), and H273Q (57.14%). Additionally, several other amino acid substitutions reached high frequency within this season, among which T120A (57.14%), N129D (85.71%), and S185I (85.71%). The substitutions P83S and D187A were predicted to exert deleterious effect on the HA1 function (score −2.603 and −3.119, respectively).

**TABLE 4:** Amino acid substitutions identified in HAs of AH1N1pdm viruses sequenced from 2016 to 2020 influenza seasons (without signal peptide).

Reference strain		AA substitutions	Antigenic site	2016/2017 (%)	2017/2018 (%)	2018/2019 (%)	(2019/2020 (%)
		S74R		0	100	100	100
		**P83S**		100	100	100	100
		S84N		100	100	100	100
		D97N		100	100	100	100
		T120A		0	81.82	16.67	57.14
		N129D		0	9.09	57.14	85.71
		K130N		0	0	0	35.71
		E154K	Sa	100	100	100	100
		S162N	Sa	100	100	100	100
	HA1	K163Q	Sa	100	100	100	100
		S164T	Sa	0	100	100	100
		S183P		0	18.18	97.62	100
		S185T	Sb	100	90.91	38.10	14.29
		S185I	Sb	0	9.09	61.90	85.71
		**D187A**	Sb	0	0	0	64.29
		Q189E	Sb	0	0	0	64.29
A/California/07/2009		S203T	Ca1	100	100	100	100
		I216T		100	100	100	100
		L233I		0	0	7.14	0
		A256T		100	100	100	100
		N260D		0	9.09	59.52	100
		H273Q		0	0	0	57.14
		K283E		100	100	100	100
		I295V		0	100	100	100
		I321V		100	100	100	100
	
		E374K (47K)		100	100	100	100
		I404M (I77M)		0	0	9.52	0
		S451N (S124N)		100	100	100	100
	HA2	E499K (E172K)		100	100	100	100
		K504R (K177R)		0	0	9.52	0
		E506D (E179D)		0	0	7.14	0
		V520A (V193A)		0	0	9.52	14.29

*Amino acid substitutions of AH1N1pdm are reported in comparison to A/California/07/2009. Deleterious mutations were predicted by the online server PROVEAN and are indicated in bold.*

#### Molecular Evolution of N1 Protein

A total of 66 NA genes of A(H1N1)pdm09 viruses were analyzed in comparison to the vaccine strain A/California/07/2009. We did not detect any substitutions among the highly conserved inner shell residues, which interact directly with the sialic acids, as well as the framework residues. However, N270K (100%) and N369K (100%) substitutions were found near the catalytic sites, whereas the N248D (100%) substitution was spatially close to the framework residues. Nine distinct variations were observed at the NA antigenic sites, among which most common were I188T (100%), V264I (100%), N270K (100%), and K432E (100%). Additional substitutions in the antigenic sites were I288V (57.14%), which emerged in the 2019/2020 season and I389K and T452I with frequencies progressively increasing throughout the seasons (Supplementary Table 1). No viruses carrying the H275Y amino acid change, conferring oseltamivir resistance, were detected.

### Genetic Characterization of H3N2 Viruses

#### Molecular Evolution of H3 Protein

Complete 79 A(H3N2) HA protein sequences were analyzed and compared against vaccine strain A/Hong Kong/4801/2014 (Table 5). Herein, the amino acids were numbered using the H3 numbering scheme excluding the signal peptide. The overall HA amino acid identities among the A(H3N2) strains isolated from 2016 to 2020 compared to the A/Hong Kong/4801/014 vaccine strain were >98%. In total 30 amino acid substitutions were detected in the H3 sequences, of which 22 were located in the HA head and 8 in the stem region. We found three substitutions in both A and B antigenic sites, two in D and one in C and E epitopes. Solely one substitution was observed in the RBS–A138S, detected in 2018–2019 season (19.23%) for the first time in our study and reaching a frequency of 100% in the 2019–2020 season. Overall, only three variants were fixed throughout the analyzed seasons, whereas various substitutions gradually replaced the parental variant in subsequent seasons before becoming the dominant variant. The most prevalent substitutions in the antigenic sites during the 2016/2017 season were N121K (91.30%) and N171K (69.57%) in epitope D, N122D (65.22%) in epitope A and K160T (100%) epitope B. Minority of the viruses were also possessing I140M (21.74%), I406V (30.43%), G479E (21.74%), G484E (30.43%), and L516I (21.74%). High frequency variants in 2018/2019 season were E62G (73.08%) in epitope E, N121K (73.08%), and N171K (73.08%) in epitope D, T128A (65.38%) in epitope A, K160T (80.77%) in epitope B. Additionally, the substitution G484E (73.08%) was predicted to exert deleterious effect on the HA2 function (score −4.942). During the 2018/2019 season various substitutions were introduced to the H3N2 virus population, such as S91N (19.23%), A138S (19.23%), Y159S (19.23%), F193S (19.23%), K326R (15.38%) M346L (19.23%), I478M (19.23%), A530V (19.23%) which predominated in the following season reaching frequencies of 86.67%. The substitution H311Q was detected only during season 2019/2020, reaching a frequency of 46.67%. In addition, substitutions T128A (epitope A) and A138S in the RSB became the dominant variant.

**TABLE 5:** Amino acid substitutions identified in HAs of H3N2 viruses sequenced from 2016 to 2020.

Reference strain	HA1	AA substitutions	Antigenic site	2016/2017 (%)	2018/2019 (%)	2019/2020 (%)
		E62G	E	0	73.08	13.33
		S91N		0	19.23	86.67
		K92R		0	73.08	13.33
		S96N		100.00	100.00	100.00
		N121K	D	91.30	73.08	13.33
		N122D	A	65.22	0	0
		T128A	A	0	65.38	100.00
		T131K		0	34.62	0
		A138S	A	0	19.23	100.00
		I140M		21.74	0	0
		R150G		0	100.00	100.00
		S144K		69.57	19.23	86.67
A/Kong/4801/2014Hong		Y159S	B	0	19.23	86.67
		K160T	B	100.00	80.77	13.33
		N171K	D	69.57	73.08	13.33
		F193S		0	19.23	100.00
		P194L		100.00	100.00	100.00
		S198P	B	0	3.85	16.67
		S262N		65.22	0	0
		K264E		0	0	0
		H311Q	C	0	0	46.67
		K326R		0	15.38	86.67
	
		M346L (M17L)		0	19.23	86.67
		I406V (I77V)		30.43	73.08	13.33
		I478M (I149M)		0	19.23	86.67
		G479E (G150E)		21.74	0	0
	HA2	**G484E (G155E)**		30.43	73.08	13.33
		L516I (L187I)		21.74	0	0
		V529I (V200I)		0	26.92	0
		A530V (A201V)		0	19.23	86.67

*Amino acid substitutions of H3N2 are reported in comparison to A/Hong Kong/4801/2014. Deleterious mutations were predicted by the online server PROVEAN and are indicated in bold.*

#### Molecular Evolution of N2 Protein

The NA gene of the A(H3N2) viruses was highly variable with a total of 31 substitutions detected in isolates from the analyzed seasons compared to the vaccine strain A/Hong Kong/4801/2014 (Supplementary Table 2). The catalytic and framework sites of the NA were highly conserved and we did not detect any variations. However, three substitutions were found near the NA active sites: Y155H, I176M, and S245N. The prevalence of Y155H was highest in season 2019/2020–86.67%, while the I176M variation was detected only in season 2018/2019 reaching a frequency of 40%. The S245N substitution was fixed throughout the four analyzed seasons. Importantly, none of the analyzed strains had the oseltamivir resistance sites E119I or R292K.

#### Genetic Characterization of Influenza B Viruses

Eighteen complete B/Yamagata HA and NA sequences and 22 B/Victoria HA and NA sequences were analyzed and compared against vaccine strain B/Phuket/3073/2013 and B/Colorado/06/2017, respectively (Table 6). The amino acids were numbered excluding the signal peptide. In total, 10 amino acid changes were found in the HA protein and none in the NA protein of the analyzed influenza B viruses. Twenty-two viruses from season 2017/2018 were sequenced, however, no amino acid changes in the antigenic sites or the RBS were discovered. The B/Victoria viruses were characterized by a double amino acid. Of the 18 B/Victoria sequenced samples during season 2019/2020, we found four amino acid substitutions with high frequency: G132R (77.78%), K135E (77.78%), V177I (77.78%), and K495R (83.3%). The most notable difference between the B/Victoria viruses from 2017/2018 vis-à-vis the 2019/2020 season viruses was the double amino acid deletion and the triple amino acid deletion, respectively.

**TABLE 6:** Amino acid substitutions identified in HAs of influenza B/Victoria and B/Yamagata during the 2017/2018 and 2019/2020 season.

Reference strain	AA substitutions	2017/2018 (%)	2019/2020 (%)
**B/Victoria**			
	E127K	75	16.67
	G128N	0	44.44
	G128D	0	38.89
	G132R	0	77.78
B/Colorado/06/2017	K135E	0	77.78
	V177I	0	77.78
	N230S	0	44.44
	K495R	0	83.33

**B/Yamagata**			
B/Phuket/3073/2013	L171Q	100	0
	M250V	100	0

*Amino acid substitutions of B/Victoria were reported in comparison to B/Colorado/06/2017, whereas the amino acid substitutions of B/Yamagata were reported in comparison to B/Phuket/3073/2013.*

## Discussion

We compared the relative contribution of influenza types A and B and subtypes H1N1pdm09, H3N2, B/Yamagata and B/Victoria *via* the ILI and SARI sentinel surveillance system and investigated the seasonality patterns in N. Macedonia throughout four consecutive seasons (2016–2020). In general, the seasonality of influenza circulation and the circulating viruses in our study was in line with the northern hemisphere with a typical peak placed between December and the middle of April. By comparing the sentinel surveillance system to the routine surveillance system, we showed that the newly applied system works efficiently and gives great results in the selection of cases. Furthermore, due to the implementation of the SARI surveillance system the number of SARI samples sent for testing notably increased from 2017/2018 season to 2019/2020 season. Possible reason of such increase is the stabilization of the SARI surveillance system in the country through the years, as well as an increased number of tested pediatric SARI cases due to the improved pediatricians’ case management of SARI cases among children age 0–4. Regarding the ILI cases, statistically significant differences (*p* = < 0.0000001) were observed when we compared the number of reported ILI cases among patients aged 0–4, 5–14, 15–29, and 30–64 years to the reference age group (65+). Additionally, we divided the two study populations and compared the number of positive ILI and SARI samples with the predominant virus type of each season. The intention was to investigate the possible connection between a particular virus subtype and the severity of the disease, however, no such distinction was made. Namely, the percentage of positive samples from the ILI and SARI surveillance was similar for all subtypes throughout the analyzed seasons. A limitation of the study is the relatively low number of positive samples per season, thus underpowering the statistical analysis when assessing the association between disease state and influenza subtype.

As a further step, we carried out an extensive phylogenetic analysis and molecular characterization of the surface glycoprotein HA of the influenza A subtypes. The phylogenetic analysis of the HA sequences unveiled a resemblance of mutations circulating seasonally worldwide, thus leading to specific clustering patterns. In accordance with published data, the vast majority of circulating viruses after 2014 belonged to the HA phylogenetic subclade 6B.1A which evolved from clade 6B.1 (Komissarov et al., 2016; Korsun et al., 2017; Puig-Barberà et al., 2017; World Health Organization, 2018, 2020; Mohebbi et al., 2019). During the 2018–2019 influenza season within the 6B.1A subclade, several genetic clusters of viruses emerged encoding a range of HA1 amino acid substitutions, with the major groups being 6B.1A2, 6B.1A5, 6B.1A6, 6B.1A7. The H3N2 viruses predominantly clustered in 3C.2a clade or the 3C.2a1 subclade and 3C.3a, as reported in other countries (World Health Organization, 2018, 2020). Since the emergence of influenza A(H1N1)pdm09 in 2009, the genetic makeup of the virus has been changing and acquiring new mutations in order to adapt to new hosts and survive in different geographic and seasonal conditions (Goka et al., 2014). Thereby, the number of HA substitutions has been increasing cumulatively and almost in every season a new phylogenetic group or subclade of influenza A(H1N1)pdm09 has been detected since 2009 (Centers for Disease Control and Prevention, 2016; World Health Organization, 2016). The alignment of the A(H1N1)pdm09 HA sequences showed a high homology between the viruses circulating during influenza seasons 2016–2020 and the vaccine strain A/California/07/2009. A shortcoming of our study was the small number of samples (*n* = 1) sequenced from season 2016/2017, thus the obtained results are not representative for the season. The distribution of substitutions in the major antigenic sites was similar to findings in other studies: E154K, S162N, K163Q, and S164T in Sa, S203T in Ca1 and S185T, S185I, D187A, and Q189E in Sb (Rudneva et al., 2012; Tsibane et al., 2012; Yasugi et al., 2013; Broberg et al., 2016; Pebody et al., 2016; Clark et al., 2017; Friedman et al., 2017; Korsun et al., 2017; Hashem et al., 2018; Liu et al., 2018; Mohebbi et al., 2019). Almost all of the amino acid residues within the RBS were highly conserved, detecting only one (D187A) substitution in the 190-loop, which is recognized in particular for its potential role in the emergence of escape mutants (Rudneva et al., 2012; Tsibane et al., 2012; Yasugi et al., 2013; Liu et al., 2018; Suntronwong et al., 2021). The D187A substitution was first detected in the 2019/2020 season and reached a frequency of 64.29% among our isolates. Moreover, the PROVEAN analysis showed that the D187A substitution has a deleterious effect on the HA function. The variant S185T/I located near the 190-helix domain of the receptor-binding site, as shown previously, can influence the antigenic properties of influenza A(H1N1)pdm09 viruses (Koel et al., 2013). In accordance with our data, more recent A(H1N1)pdm09 strains have acquired substitutions S74R, S164T, and I295V, which have evolved into subclade 6B.1A (World Health Organization, 2019). The emergence of S183P adjacent to the Sb antigenic site forms subclades 6B.1A1 to 6B.1A7 (World Health Organization, 2019) and some studies have shown that serum from those immunized with the vaccine strain of clade 6B.1 was unable to efficiently neutralize influenza virus strain carrying S183P (Epperson et al., 2019). Our analysis showed that the frequency of S183P was significantly higher (*p* = 0.00001) in season 2018/2019 (97.62%) vis-a-vis season 2017/2018 (18.18%). Notably, we did not detect the D222G/N mutation in the HA gene of influenza A(H1N1)pdm09 which may lead to an increase in viral replication in the lower respiratory tract and a worsening of clinical conditions (Kilander et al., 2010; Kuroda et al., 2010; Wedde et al., 2013). H3N2 viruses acquire mutations rapidly. The evolutionary rate for A(H3N2) in the HA1 domain is considerably greater than that for A(H1N1)pdm09 (Kirkpatrick et al., 2018). Since 2009, the number of fixed mutations has increased dramatically from one fixed substitution in 2009 to 15 in 2017 (Al Khatib et al., 2019). We detected various substitutions which gradually replaced the parental variant in subsequent seasons before becoming the dominant variant. The viruses carrying the RBS substitution, A138S (epitope A), fell into the subgroup 3C.3a and studies showed this variant leads to increase in the receptor binding avidity (Chambers et al., 2015). Since 2017, 3C.2a1b was established, which has gained additional substitutions (either T131K or T135K with T128A) in HA1 and these changes are located in the antigenic and the RBS region (Broberg et al., 2016). The substitutions T128A and T131K were first detected in our isolates in season 2018/2019 and T128A predominated in the following season. T128A, which is located at antigenic site A, is predicted to cause a loss of glycosylation that might alter the HA antigenic properties and affect antibody recognition (Zost et al., 2017). Additionally, the T128A mutation had been previously reported to be associated with high mortality rate among children (Bragstad et al., 2008). Variant F193S was first introduced in 2018/2019 and became the dominant variant in the next season. The residue at position 193 is located on a highly exposed region within the 190-helix in RBS and changes at this residue likely impact antibody recognition and promote escape (Jorquera et al., 2019). During 2015, several novel mutations were acquired at the major antigenic sites: N121K, K160T, N171K (Al Khatib et al., 2019) and accordingly they were detected in our samples as well. However, the frequencies of these substitutions decreased dramatically in the subsequent years. Of note, the substitution G484E was predicted to exert deleterious effect on the HA2 function (score −4.942), as shown in a study by Al Khatib et al. (2019).

The analysis of the protein sequences of influenza B viruses did not discover any amino acid changes in the antigenic sites or the RSB. New antigenic variants of A(H3N2) viruses appear every 3–5 years, whereas new antigenic variants of A(H1N1)pdm09 and influenza B viruses are less frequent (Smith et al., 2004; Chen and Holmes, 2008; Bedford et al., 2015; Vijaykrishna et al., 2015). Therefore, a continuous and widespread monitoring scheme of influenza A(H1N1)pdm09 and A(H3N2) viruses is necessary for the early detection of newly emerging variants with pandemic potential, identification of novel strains which may alter the effectiveness of available vaccination, determination of the genetic basis of pathogenicity, antiviral resistance and an ultimately better understanding of viral evolution. With the introduction of the sentinel surveillance, accompanied by the advances made in rapid whole-genome sequencing and drug and vaccine therapeutics, public health officials can now modify their approach to disease management and intervene more effectively as well as in a timely manner in order to prevent major morbidity and mortality from influenza. In North Macedonia, the results obtained in the laboratory for the ILI and SARI cases are immediately available to the epidemiologists, allowing them in real time to assess the epidemiological situation and to recommend public health and social measures, in order to contain and control the disease from further spreading. Additionally, the out-season surveillance can mark an earlier start of the influenza season or hint the circulating subtype of the upcoming season, allowing early evaluation of the vaccine efficiency. Moreover, the obtained data for the genetic characterization of the influenza viruses summarized in this study is uploaded in GISAID, making it available to the international public health authorities and allowing continuous monitoring of the circulating variants. Recent efforts have made progress in predicting influenza virus genetic evolution by analyzing genetic sequence data and phylogenetic patterns to predict the success of genetic lineages (Łuksza and Lässig, 2014; Neher et al., 2014). The present work is the first molecular study characterizing the influenza viruses circulating in North Macedonia during 2016–2020, which contributes to better managing, controlling, and limiting of future influenza outbreaks and pandemics.

## Data Availability Statement

Accession numbers of A(H1N1)pdm09 and A(H3N2) representative strains used for phylogenetic analysis as obtained from GISAID (http://www.gisaid.org) (Supplementary Table 3).

## Author Contributions

MK, GB, SM, and IG contributed to the conception and design of the study. GK, DK, KS, VM, and RSI organized the database. DK, RSI, and MK performed the statistical analysis. MK, TB, EJ, MS, AP, and GN carried out the laboratory analyses. MK wrote the manuscript. All authors contributed to manuscript revision, read, and approved the submitted version.

## Conflict of Interest

The authors declare that the research was conducted in the absence of any commercial or financial relationships that could be construed as a potential conflict of interest.

## Publisher’s Note

All claims expressed in this article are solely those of the authors and do not necessarily represent those of their affiliated organizations, or those of the publisher, the editors and the reviewers. Any product that may be evaluated in this article, or claim that may be made by its manufacturer, is not guaranteed or endorsed by the publisher.

## Footnotes

1
http://www.gisaid.org

2
https://usegalaxy.eu/

3
http://provean.jcvi.org

## Supplementary Material

Supplementary Figure 1 Graphical comparison between the routine and sentinel surveillance in North Macedonia.

Supplementary Figure 2 Graphical representation of reported ILI cases from 2017 to 2020 divided by age groups.

Supplementary Figure 3 Comparison of the number of reported, tested and positive SARI cases throughout the four analyzed season.
