# Supplementary material for: A Comprehensive Molecular and Epidemiological Characterization of Influenza Viruses Circulating 2016–2020 in North Macedonia
Source: Front Microbiol. 2021 Oct 21;12:713408. doi: 10.3389/fmicb.2021.713408 (PMC8567633; doi:10.3389/fmicb.2021.713408)
Supplement: Supplementary Figure 1 — Graphical comparison between the routine and sentinel surveillance in North Macedonia. [file Presentation_1.pdf]

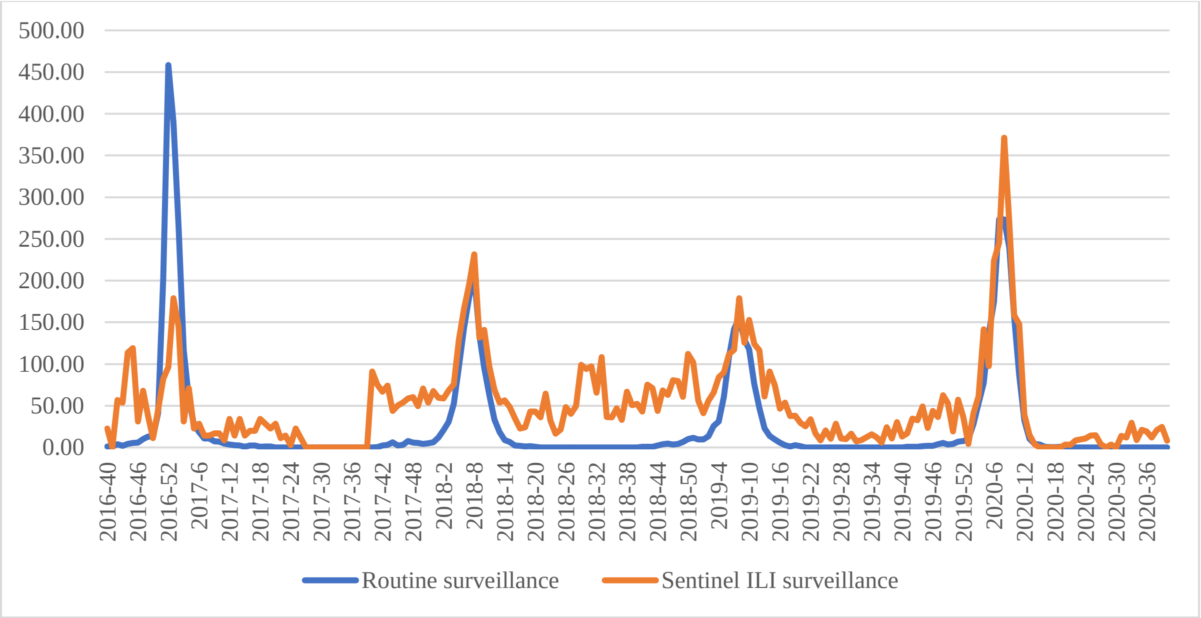

Supplementary figure 1. Graphical comparison between the routine and sentinel surveillance in North Macedonia
