# Supplementary material for: A Comprehensive Molecular and Epidemiological Characterization of Influenza Viruses Circulating 2016–2020 in North Macedonia
Source: Front Microbiol. 2021 Oct 21;12:713408. doi: 10.3389/fmicb.2021.713408 (PMC8567633; doi:10.3389/fmicb.2021.713408)
Supplement: Supplementary Figure 2 — Graphical representation of reported ILI cases from 2017 to 2020 divided by age groups. [file Presentation_2.pdf]

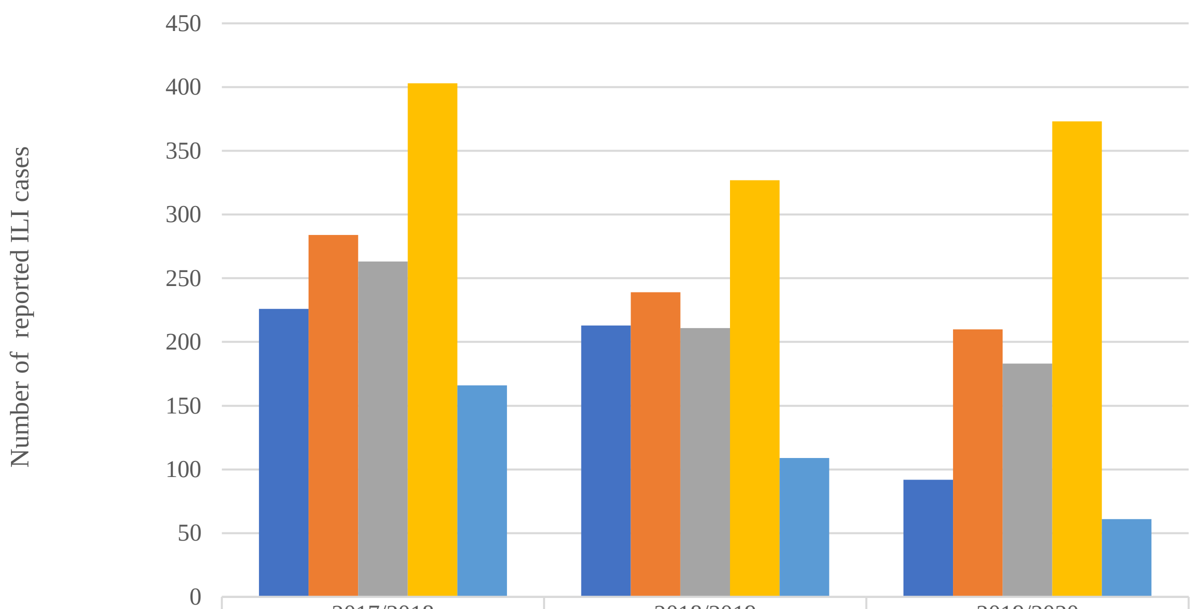

	2017/2018	2018/2019	2019/2020
Sum of ILI_0-4	226	213	92
Sum of ILI_5-14	284	239	210
Sum of ILI_15-29	263	211	183
Sum of ILI_30-64	403	327	373
Sum of ILI_65+	166	109	61

Supplementary figure 2. Graphical representation of reported ILI cases from 2017 to 2020 divided by age groups
