# Supplementary material for: A Comprehensive Molecular and Epidemiological Characterization of Influenza Viruses Circulating 2016–2020 in North Macedonia
Source: Front Microbiol. 2021 Oct 21;12:713408. doi: 10.3389/fmicb.2021.713408 (PMC8567633; doi:10.3389/fmicb.2021.713408)
Supplement: Supplementary file 4 [file Data_Sheet_1.docx]

Supplementary table 1. Amino acid substitutions identified in NAs of A(H1N1)pdm09 viruses sequenced during 2016-2020. Amino acid substitutions of A(H1N1)pdm09 are reported in comparison to A/California/07/2009.

| Reference strain | AA substitutions | 2016/2017 (%) | 2017/2018 (%) | 2018/2019 (%) | (2019/2020 (%) |
| --- | --- | --- | --- | --- | --- |
| A/California/07/2009 | I34V | 100 | 100 | 100 | 100 |
|  | L40I | 100 | 100 | 100 | 100 |
|  | N44S | 100 | 100 | 100 | 100 |
|  | N50I | 0 | 0 | 22.5 | 0 |
|  | Q51K | 0 | 9.09 | 60 | 100 |
|  | V62I | 0 | 27.27 | 5 | 0 |
|  | T72I | 0 | 81.82 | 17.50 | 14.29 |
|  | F74L | 0 | 0.00 | 10 | 0 |
|  | F74S | 0 | 9.09 | 55 | 100 |
|  | G77R | 0 | 100 | 100 | 100 |
|  | V81A | 0 | 100 | 100 | 100 |
|  | I188T | 0 | 100 | 100 | 100 |
|  | N200S | 100 | 100 | 100 | 100 |
|  | V241I | 100 | 100 | 100 | 100 |
|  | N248D | 100 | 100 | 100 | 100 |
|  | V264I | 100 | 100 | 100 | 100 |
|  | N270K | 100 | 100 | 100 | 100 |
|  | I288V | 0 | 0 | 0 | 57.14 |
|  | I314M | 100 | 100 | 100 | 100 |
|  | I321V | 100 | 100 | 100 | 100 |
|  | I365T |  | 54.55 | 15 | 0 |
|  | N369K | 100 | 100 | 100 | 100 |
|  | N386K | 100 | 100 | 100 | 100 |
|  | I389K | 0 | 9.09 | 37.5 | 100 |
|  | D416N | 0 | 81.82 | 75 | 100 |
|  | K432E | 100 | 100 | 100 | 100 |
|  | I436V | 0 | 0 | 10 | 0 |
|  | N449D | 0 | 100 | 100 | 100 |
|  | D451G | 0 | 36.36 | 0 | 0 |
|  | T452I | 0 | 9.09 | 60 | 100 |

Supplementary table 2. Amino acid substitutions identified in NAs of A(H3N2) viruses sequenced from 2016-2020. Amino acid substitutions of H3N2 are reported in comparison to A/Hong Kong/4801/2014.

| Reference strain | Antigenic site | 2016/2017 (%) | 2018/2019 (%) | 2019/2020 (%) |
| --- | --- | --- | --- | --- |
| A/Hong Kong/4801/2014 | N38K | 0.00 | 0.00 | 13.33 |
|  | I57M | 0.00 | 4.00 | 86.67 |
|  | E64K | 0.00 | 28.00 | 0.00 |
|  | K75R | 0.00 | 20.00 | 86.67 |
|  | I77V | 0.00 | 20.00 | 0.00 |
|  | G93D | 69.57 | 0.00 | 0.00 |
|  | P126L | 0.00 | 40.00 | 13.33 |
|  | L140I | 8.70 | 20.00 | 86.67 |
|  | V149A | 8.70 | 20.00 | 86.67 |
|  | Y155H | 0.00 | 20.00 | 86.67 |
|  | N161S | 69.57 | 20.00 | 86.67 |
|  | I176M | 0.00 | 40.00 | 0.00 |
|  | I194V | 0.00 | 28.00 | 0.00 |
|  | K220N | 0.00 | 40.00 | 13.33 |
|  | I231V | 100.00 | 100.00 | 100.00 |
|  | S245N | 100.00 | 100.00 | 100.00 |
|  | S247T | 100.00 | 100.00 | 100.00 |
|  | T267K | 100.00 | 100.00 | 100.00 |
|  | V303I | 0.00 | 40.00 | 13.33 |
|  | S315R | 0.00 | 28.00 | 0.00 |
|  | N329S | 21.74 | 80.00 | 13.33 |
|  | N329T | 0.00 | 20.00 | 86.67 |
|  | S331R | 0.00 | 28.00 | 0.00 |
|  | D339N | 100.00 | 100.00 | 100.00 |
|  | E344K | 0.00 | 48.00 | 100.00 |
|  | G346V | 65.22 | 0.00 | 0.00 |
|  | I380V | 100.00 | 72.00 | 100.00 |
|  | P386S | 0.00 | 60.00 | 86.67 |
|  | T392I | 100.00 | 100.00 | 100.00 |
|  | P468L | 61.54 | 0.00 | 0.00 |
|  | P468H | 30.43 | 100.00 | 100.00 |

Supplementary table 3. Accession numbers of A(H1N1)pdm09 and A(H3N2) representative strains used for phylogenetic analysis as obtained from GISAID.

| **Representative reference strains used in phylogeny** | | | | | |
| --- | --- | --- | --- | --- | --- |
| EPI_ISL_331393 | EPI_ISL_338060 | EPI_ISL_315812 | EPI_ISL_342128 | EPI_ISL_387064 | EPI_ISL_291668 |
| EPI_ISL_309971 | EPI_ISL_299047 | EPI_ISL_294113 | EPI_ISL_300473 | EPI_ISL_292849 | EPI_ISL_387060 |
| EPI_ISL_367924 | EPI_ISL_394081 | EPI_ISL_394054 | EPI_ISL_348116 | EPI_ISL_262256 | EPI_ISL_991692 |
| EPI_ISL_943262 | EPI_ISL_913773 | EPI_ISL_516528 | EPI_ISL_166859 | EPI_ISL_256072 | EPI_ISL_959871 |
| EPI_ISL_959871 | EPI_ISL_340478 | EPI_ISL_410633 | EPI_ISL_344427 | EPI_ISL_206099 | EPI_ISL_332840 |
| EPI_ISL_73686 | EPI_ISL_68771 | EPI_ISL_68771 | EPI_ISL_90787 | EPI_ISL_299017 | EPI_ISL_299034 |
| EPI_ISL_165553 | EPI_ISL_127832 | EPI_ISL_262230 | EPI_ISL_259578 | EPI_ISL_340478 | EPI_ISL_410633 |
| EPI_ISL_344427 | EPI_ISL_206099 | EPI_ISL_332840 | EPI_ISL_331393 | EPI_ISL_338060 | EPI_ISL_315812 |
| EPI_ISL_342128 | EPI_ISL_387064 | EPI_ISL_291668 | EPI_ISL_477546 | EPI_ISL_477546 | EPI_ISL_342005 |
| EPI_ISL_377296 | EPI_ISL_312041 | EPI_ISL_344423 | EPI_ISL_344423 | EPI_ISL_292377 | EPI_ISL_330891 |
| EPI_ISL_364801 | EPI_ISL_406276 | EPI_ISL_486701 | EPI_ISL_434407 | EPI_ISL_529043 | EPI_ISL_498387 |
| EPI_ISL_477527 | EPI_ISL_455556 | EPI_ISL_485720 | EPI_ISL_314925 | EPI_ISL_389025 | EPI_ISL_410605 |
| EPI_ISL_377321 | EPI_ISL_162145 | EPI_ISL_314181 |  |  |  |
